# A novel immunotoxin reveals a new role for CD321 in endothelial cells

**DOI:** 10.1371/journal.pone.0181502

**Published:** 2017-10-13

**Authors:** Takeshi Fukuhara, Jia Kim, Shintaro Hokaiwado, Makiko Nawa, Hayato Okamoto, Tomohiko Kogiso, Tetsuro Watabe, Nobutaka Hattori

**Affiliations:** 1 Department of Neurology, Juntendo University School of Medicine, Bunkyo-ku, Tokyo, Japan; 2 Laboratory of Oncology, Department of Life Science, Tokyo University of Pharmacy and Life Sciences, Hachioji city, Tokyo, Japan; 3 Department of Biochemistry, Graduate School of Medical and Dental Sciences, Tokyo Medical and Dental University, Bunkyo-ku, Tokyo, Japan; 4 Laboratory of Cytometry and Proteome Research in Medical Research Institute, Tokyo Medical and Dental University, Bunkyo-ku, Tokyo, Japan; Duke University School of Medicine, UNITED STATES

## Abstract

There are currently several antibody therapies that directly target tumors, and antibody-drug conjugates represent a novel moiety as next generation therapeutics. Here, we used a unique screening probe, DT3C, to identify functional antibodies that recognized surface molecules and functional epitopes, and which provided toxin delivery capability. Accordingly, we generated the 90G4 antibody, which induced DT3C-dependent cytotoxicity in endothelial cells. Molecular analysis revealed that 90G4 recognized CD321, a protein localized at tight junctions. Although CD321 plays a pivotal role in inflammation and lymphocyte trans-endothelial migration, little is known about its mechanism of action in endothelial cells. Targeting of CD321 by the 90G4 immunotoxin induced cell death. Moreover, 90G4 immunotoxin caused cytotoxicity primarily in migratory endothelial cells, but not in those forming sheets, suggesting a critical role for CD321 in tumor angiogenesis. We also found that hypoxia triggered redistribution of CD321 to a punctate localization on the basal side of cells, resulting in functional impairment of tight junctions and increased motility. Thus, our findings raise the intriguing possibility that endothelial CD321 presented cellular localization in tight junction as well as multifunctional dynamics in several conditions, leading to illuminate the importance of widely-expressed CD321 as a potential target for antitumor therapy.

## Introduction

Several types of monoclonal antibodies, with different mechanisms of action, are clinically available for targeted cancer therapy [1]. In addition to major pathways, such as complement-dependent cytotoxicity and antibody-dependent cellular cytotoxicity, some functional antibodies exhibit remarkable therapeutic effectiveness by modulating specific signaling cascades such as the vascular endothelial growth factor (VEGF) pathway during tumor angiogenesis [2]. Furthermore, antibody-drug conjugates (ADC) face increasing demand as they can minimize both medication dose and severity of side effects. The discovery of specific and functional antibodies capable of delivering drugs into target cells remains a challenge. To be eligible for a functional component of ADC, an antibody must bind strongly to target cells and need to be internalized.

To overcome the limitations regarding the discovery of ADC-compatible antibodies, we developed unique probes for hybridoma screening, such as FZ33-Adv [3,4] and DT3C [5,6]. DT3C encodes a diphtheria toxin lacking the receptor- and Fc-binding domains derived from *Streptococcus* protein G. In principle, DT3C should exhibit cytotoxicity only if the immunocomplex formed with the antibody is internalized. Recombinant DT3C proteins enabled us to evaluate whether an antibody of interest was internalized by specific cells. We exploited the potential of the DT3C immunotoxin assay in conjunction with conventional hybridoma technology to screen a hybridoma library for ADC-compatible monoclonal antibodies. Because an immunotoxin assay uses live cells, it is necessary for potent antibodies to recognize the native structure of the antigen on the cell surface. Moreover, sufficient functionality and epitope specificity are required for immunotoxins exhibiting DT3C-dependent cytotoxicity.

On the basis of this information, we aimed to identify prospective molecules specifically that are expressed in the stromal cells of the tumor microenvironment. For this purpose, we conducted a screening of functional antibodies compatible with ADC properties, aimed in particular at endothelial cells critical for tumor growth and metastasis.

Herein, we present evidence that the CD321 molecule recognized by the 90G4 antibody plays a critical role in endothelial cell migration and angiogenesis. CD321 is expressed in platelet, leukocyte, and endothelial cells. As CD321 can bind to the lymphocyte function-associated antigen 1 (LFA1) expressed on leukocytes, CD321-deficient animals present reduced infiltration of neutrophils, resulting in attenuation of inflammation [7–9]. Knowing that CD321 plays pivotal roles in tumor metastasis, it may be attractive to target CD321 molecule using specific antibodies for potential anti-tumor therapy.

## Materials and methods

### Reagents and plasmids

Tetrazolium salts WST-1 and 1-methoxy PMS were purchased from Dojindo (Kumamoto, Japan). Luminol and *p*-coumaric acid were purchased from Sigma (St. Louis, MO, USA). The pCMV-SPORT6 expression vector (BC021876) encoding mouse CD321 was obtained from TransOMIC technologies (Huntsville, AL, USA). The pCMV6-C-terminal Myc-DDK-tagged ORF clones of mouse CD322 (Jam2, MR204155) and CD323 (Jam3, MR204404) were purchased from OriGene Technologies, Inc. (Rockville, MD USA).

### Cell culture

Endothelial MS-1 [10], SVEC4-10 [11], and b.End3 [12], and epithelial CHO cells were obtained from the American Type Culture Collection (Rockville, MD, USA). Endothelial F-2 cells [13] was a gift from Dr. H. Yagita. Endothelial TKD2 cells (IFO50374) [14] was purchased from JCRB cell bank (Ibaraki, Osaka, JAPAN). Cells were cultured in alpha-MEM (Nacalai, Kyoto, Japan) for MS-1 cells, or in DMEM (ibid.) for SVEC4-10, b.End3 and F-2 cells, or in F-12 (ibid.) for CHO cells. Growth media contained 50 U mL^−1^ penicillin, 50 U mL^−1^ streptomycin (both Nacalai, Kyoto, Japan), and 10% fetal bovine serum (FBS, HyClone, Logan, UT, USA). For TKD2 cells, VascuLife VEGF Comp kit (Kurabo, Osaka, JAPAN) was used in this study. Hypoxia (pO_2_ = 1%) or normoxia (pO_2_ = 21%) was maintained at 5% CO_2_ and 37°C in a multi-gas incubator system (9000EX; Wakenyaku Co ltd, Kyoto, Japan) and a CO_2_ incubator (MCO-175; Sanyo, Moriguchi, Japan).

### Hybridoma production and antibody purification

The following experimental protocol used in this study was approved by animal experiment committee in Tokyo University of Pharmacy and Life Sciences (L15-12). Swiss rats (SLC, Shizuoka, Japan) aged 7–10 weeks were immunized weekly for at least five weeks with 5 × 10^6^ MS-1 cells by intra peritoneal injection, following boost immunization three days prior to sacrifice animal by dislocation or injecting Pentobarbital (Kyoritsu seiyaku corporation, Tokyo, Japan) for collecting spleen. Immunized splenocytes and NSO^bcl2^ myeloma cells [15] (kindly provided by Dr. B. Diamond upon signing a material transfer agreement) were fused with PEG1500 (Roche, Basel, Switzerland). The hybridoma library was incubated for about a week in HAT selection medium containing Super Low IgG FBS (HyClone), after which the immunotoxin assay was performed (see section below). To clone the hybridoma, positive wells in the library were subjected to limiting dilution. The immunoglobulin (Ig) subclass was determined using a rat monoclonal antibody isotyping test kit (Bio-Rad, Hercules, CA, USA) following the manufacturer’s instructions. For antibody purification, the culture supernatant was diluted 1:2 in protein G IgG binding buffer (Thermo Scientific, Waltham, MA, USA), followed by capture with COSMOGEL Ig-Accept protein G (Nacalai). Protein G IgG elution buffer was used for elution, followed immediately by neutralization with 0.1 M Tris-HCl pH 9.0. Fractions containing 90G4 antibodies were dialyzed against 0.9% NaCl (Slide-A-Lyzer dialysis cassettes with molecular weight cutoff of 10K, 66380; Thermo Scientific), prior to sterilization through 0.45-μm cellulose acetate filters (DISMIC-03CP; Advantec, Tokyo, Japan). Antibody concentration was determined using the NanoDrop 1000 (Thermo Scientific).

### Immunotoxin assay

Recombinant DT3C was purified as previously described [5] with minor modifications. Briefly, to minimize nonspecific effects of DT3C, we used culture medium containing Super Low IgG FBS. The indicated amounts of DT3C and purified antibody were pre-incubated for at least 30 min in a CO_2_ incubator, then immunocomplexes and 10^4^ SVEC4-10 cells were seeded simultaneously in 96-well plates. After three days of cultivation in a CO_2_ incubator, cell viability was measured with the WST-1 reagent (5 mM WST-1, 2 mM 1-methoxy PMS, 18 mM HEPES pH 7.4). Absorbance at 460 nm and 650 nm (as reference) was measured using a plate reader (Benchmark Plus; Bio-Rad) and relative cell viability was calculated.

### Flow cytometry

For flow cytometry analysis, 2.5 × 10^5^ cells were typically stained with 2.5 μg of primary antibodies: 90G4 (prepared in-house) or isotype control (IgG2a,к; BioLegend, San Diego, CA, USA). Anti-IgG (H+L chain) FITC-conjugated rat polyclonal antibody (MBL, Nagoya, Japan) was employed as secondary antibody. Live cells were dissociated with TripLE reagent (Invitrogen, Carlsbad, CA, USA), stained with primary and secondary antibodies for 30 min each on ice, washed with PBS (-), and measured on a FACS Calibur (BD Biosciences, San Diego, CA, USA). For data analysis and visualization we used FlowJo software (FlowJo, LLC, Ashland, OR, USA).

To confirm immunoreactivity of the 90G4 antibody, a murine CD321 expression plasmid was transfected with Lipofectamine 2000 (Thermo Scientific) into CHO cells. Cells were analyzed by flow cytometry two days after transfection.

### Biotinylation and immunoprecipitation

To biotinylate surface proteins, approximately 5 × 10^7^ cells on a culture dish were labeled with 0.2 mg mL^−1^ EZ link Sulfo-NHS Biotin (Thermo Scientific) in 10 mL PBS (-) for 30 min at room temperature on a horizontal shaker (NJ-022NS; Nisshin Rika, Tokyo Japan). To stop the reaction, cells were treated with PBS (-) containing 100 mM glycine, followed by lysis in NP40 buffer (150 mM NaCl, 50 mM Tris-HCl pH 7.6, 1% NP40, and complete Roche protease inhibitor cocktail). After incubation on ice for 30 min, the NP40-soluble fraction was isolated by centrifugation at 20,400 × *g* for 20 min. As for immunoprecipitation, the lysate was pre-cleared by incubation with Ig-Accept protein G beads to remove non-specific binding. Subsequently, the lysate was subjected to immunoprecipitation with either 90G4 or isotype control antibodies, followed by capture with Ig-Accept beads. After the binding step on ice, washes were carried out with NP40 lysis buffer without protease inhibitor. Final precipitates were directly dissolved and heat-denatured in an equivalent volume of beads in SDS sample buffer (125 mM Tris-HCl pH 6.8, 6% SDS, 40% glycerol, 0.02% bromophenol blue, and 355 mM 2-mercapto ethanol). SDS-polyacrylamide gel electrophoresis (PAGE) was performed using a 5–20% gradient gel (Nacalai). Proteins were then transferred to a PVDF membrane (Immobilon-P; Millipore, Bedford, MA, USA) with semi-dry transfer buffer (192 mM glycine, 25 mM Tris-HCl, 20% (v/v) ethanol, 0.37% SDS). Blocking was carried out with 5% skim milk (Nacalai), followed by probing with a streptavidin-HRP conjugate (GE Healthcare, Little Chalfont, Buckinghamshire, UK) overnight. To prepare the detection reagent, equivalent volumes of chemiluminescent reagent A (100 mM Tris-HCl pH 8.5, 2.5 mM luminol, 0.4 mM *p*-coumaric acid) and reagent B (0.1 M Tris-HCl pH 8.5, 0.015% H_2_O_2_) were premixed, then used to soak the PVDF membrane. Chemiluminescent images were captured with a biomolecular imager (LAS4000; GE Healthcare).

### Mass spectrometry

The immunoprecipitated samples separated by SDS-PAGE were stained with a silver staining kit (Nacalai). For in-gel digestion, proteins contained in gel pieces were carbamidomethylated using 10 mM DTT at 60°C for 1 h and subsequently blocked with 50 mM iodoacetamide at room temperature for 45 min, followed by digestion with 1 pmol of sequencing-grade trypsin (Promega, Madison, WI, USA). After multiple steps of extraction by peptide digestion, liquid chromatography-tandem mass spectrometry (LC-MS/MS) analysis using the mXis4G-CPR spectrometer (Bruker Daltonics, Leipzig, Germany) was performed in buffer-A (0.1% formic acid) and a 10–35% gradient of buffer-B (100% acetonitrile) for 55 min. Peptide mass data were submitted to an in-house database for peptide mass fingerprinting.

### Live imaging

SVEC4-10 cells (1 × 10^4^) were seeded in 96-well ImageLock plates and imaged every 3 h with the IncuCyte system (Essen BioScience, Ann Arbor, MI, USA) at atmospheric O_2_ conditions (21%) and 5% CO_2_. Three images were taken per well in triplicate samples. To calculate the percentage of confluency, a series of phase contrast images were analyzed with the IncuCyte ZOOM 2016A software (Essen BioScience). Exported datasets were presented graphically using GraphPad Prism7 (GraphPad Software, La Jolla, CA, USA).

### Immunocytochemistry and confocal microscopy

For immunocytochemistry, SVEC4-10 cells were seeded on pre-washed 0.13–17-mm-thick coverslips (1-S; Matsunami, Osaka, Japan) under either hypoxia or normoxia for three days. After fixation with 4% PFA and subsequent incubation in blocking solution (0.2% gelatin, 1% BSA, 0.05% Tween in PBS (-)), coverslips were incubated with primary antibodies diluted in blocking solution overnight at 4°C. Each coverslip was multi-color stained with 2 μg rat anti-mouse CD321 (90G4, prepared in-house) and rabbit anti-SM22α (1:5000, ab9614106; Abcam, Cambridge, UK) antibodies. As for detecting myc-tag, anti-Myc antibody (1:2000, MC045; Nacalai, Tokyo, Japan). Highly cross-absorbed Alexa488 donkey anti-rat Ig (H+L) antibody, Alexa594 donkey anti-rabbit Ig (H+L) antibody, and Alexa555 donkey anti-mouse Ig (H+L) antibody (Invitrogen) secondary antibodies were used. Specimens were mounted using ProLong Gold Antifade Reagent with DAPI (Invitrogen). Multi-color images were obtained by confocal laser microscopy (LSM780; Carl Zeiss MicroImaging, Jena, Germany), using a 100× objective (alpha Plan-Apochromat 100×/1.46 Oil DIC M27) for high magnification and fluorescent excitation at 405 nm, 488 nm, and 560 nm. Fluorescent emission signals for DAPI, Alexa488, and Alexa594 were acquired in multi-track mode through band pass filters BP410-495, BP490-552, and BP570-640, respectively. Under high magnification, the multi-track Z-stack imaging mode was used to take orthogonal images generated by the ZEN image browser. All images were taken at room temperature.

## Results and discussion

### 90G4 immunotoxin induces cell death

**Fig 1:**
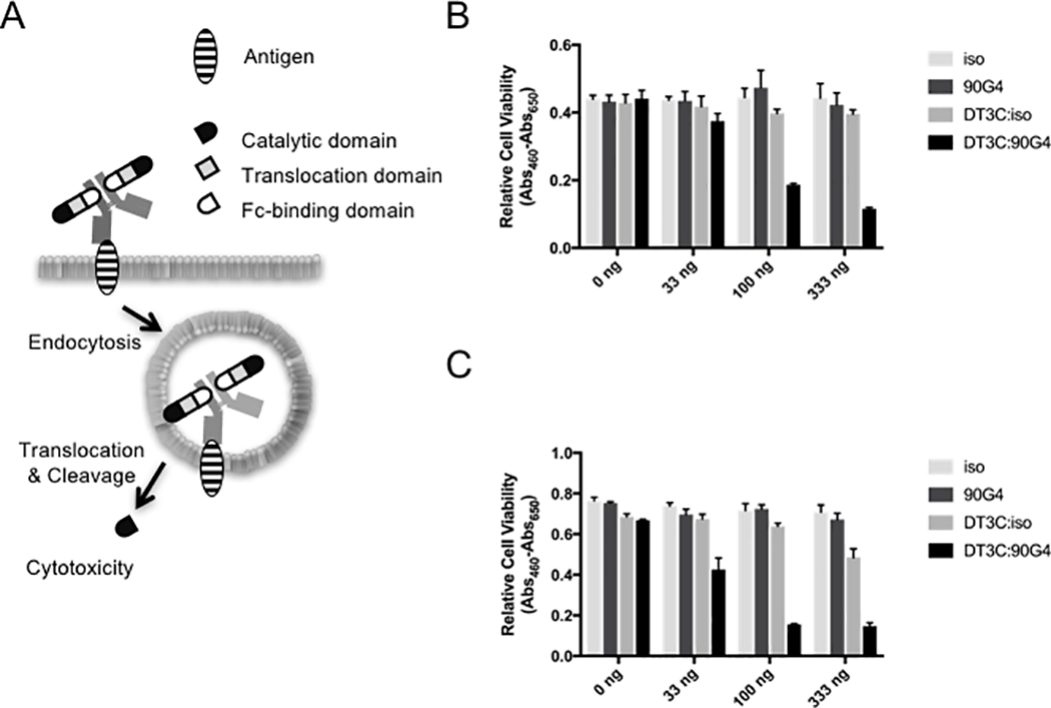
90G4 monoclonal antibody displays immunotoxin activity. (A) Principle of the DT3C-mediated immunotoxin screening. The antibody forms an immunocomplex with DT3C via the Fc-binding domain. If the immunocomplex bound to the cell surface is internalized with the target antigen, cleavage of the DT3C catalytic domain by the intracellular furin protease results in cytotoxicity. DT3C with either purified 90G4 or isotype control (iso; IgG2a, к) antibodies was administered to (B) MS-1 or (C) SVEC4-10 cells. After three days, cell viability was measured with the WST-1 assay. Data represent the mean value of triplicate samples for three independent experiments. Error bars correspond to the standard error of the mean.

To isolate potent antibody-secreting hybridomas capable of inducing DT3C-dependent cytotoxicity, we first generated a hybridoma library against MS-1 murine endothelial cells and performed the DT3C immunotoxin assay. As summarized in Fig 1A, the immunocomplex formed by DT3C and the antibody is internalized only if the potential antibody recognizes a prospective antigen expressed on the cell surface. Following internalization, DT3C is cleaved at the translocation domain by the cytosolic furin protease, releasing the catalytic domain into the cytoplasm. Subsequently, the catalytic domain of DT3C causes ADP-ribosylation of elongation factor (EF)-2, blocking the protein translation machinery and resulting in cell death. Using this principle, a potent hybridoma can be selected simply by monitoring cytotoxicity against MS-1 cells. After employing the immunotoxin assay and limiting dilution, the 90G4 monoclonal hybridoma clone was established. Purified 90G4, but not isotype control antibodies, resulted in dose-dependent DT3C-induced cytotoxicity against MS-1 (Fig 1B) and SVEC4-10 (Fig 1C) cells. Given that cytotoxicity was not observed with the 90G4 antibody alone, the effect might be attributed to antibody-antigen binding. We observed a slight cytotoxic effect suggesting non-specific uptake of DT3C at the highest concentration tested (Fig 1C). Thus, the isolated 90G4 monoclonal antibody appeared suitable for drug delivery to endothelial cells.

### The antigen recognized by the 90G4 monoclonal antibody is CD321

**Fig 2:**
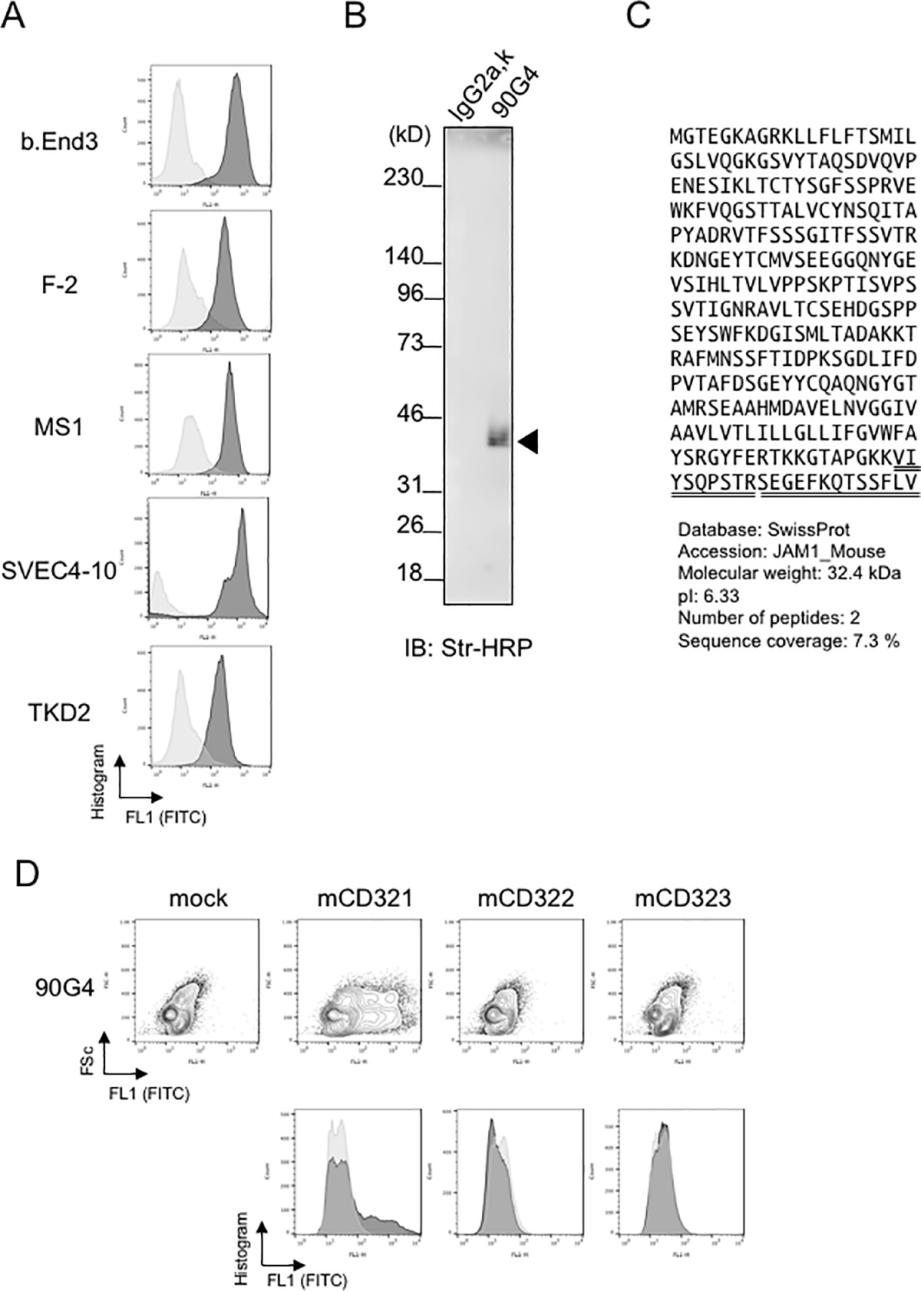
90G4 monoclonal antibody recognizes the CD321 antigen. (A) Expression profile in endothelial cell lines. Indicated endothelial cell lines were tested by staining with control (gray) or 90G4 antibodies (black). Data of FITC fluorescent intensity (FL1) indicated in histogram. Data presents three independent experiments. (B) Biochemical profiling of the molecular weight of the putative antigen. SVEC4-10 cells were surface-biotinylated with Sulfo-NHS-Biotin and lysed in NP40 buffer, followed by immunoprecipitation with 90G4 or IgG2a, к control antibodies. After SDS-polyacrylamide gel electrophoresis (PAGE), the putative antigen was visualized by probing the streptavidin-HRP (Str-HRP) conjugate by chemiluminescence. The molecular weight of the detected protein was 35 kDa. (C) Identification of 90G4 antigen. Amino acid sequence of CD321 proteins were indicated with the two of underlined peptide sequences that are detected by LC-MS/MS analysis. (D) Specific immunoreactivity of 90G4 antibody against CD321. The expression vectors encoding CD321, CD322, or CD323 cDNA were transfected into CHO cells, which were then subjected to flow cytometry analysis. Data are presented in contour plot (top) or overlaid in histogram (bottom) of FITC signal intensity of sample (black) or control (gray).

Next, we used flow cytometry to assess expression of the putative antigen recognized by the 90G4 antibody and found significant immunoreactivity against five endothelial cell lines, b.End3, F-2, MS-1, SVEC4-10, and TKD2 (Fig 2A). To identify the putative antigen, surface proteins on SVEC4-10 cells were labeled with Sulfo-NHS-Biotin and immunoprecipitated with 90G4 or isotype control antibodies. A 35-kDa band corresponding to a putative antigen (Fig 2B) was identified by immunoblotting. A similar band was also detected using the biotinylated lysate of b.End3 cells (not shown). To identify the antigen by peptide mass fingerprinting, we performed large-scale immunoprecipitation of b.End3 cell lysates. The 35-kDa target band was detected by silver staining and subjected to LC-MS/MS analysis. The sequenced peptides identified CD321 as the candidate of 90G4 antigen (Fig 2C, underlined). To validate the specific antigenicity of the 90G4 antibody, a mock or JAMs (CD321, CD322, or CD323) expression vectors were transfected into CHO cells, followed by flowcytometry analysis. The 90G4 antibody reacted with murine CD321- but not others, confirming that CD321 was a genuine antigen of the 90G4 antibody (Fig 2D). Expression of CD322 and CD323 in CHO cells was confirmed by immunocytochemical analysis for myc-tag epitopes at their carboxy termini, which were not detected by 90G4 antibody (S1 Fig).

### CD321 regulates cell spreading and migration

**Fig 3:**
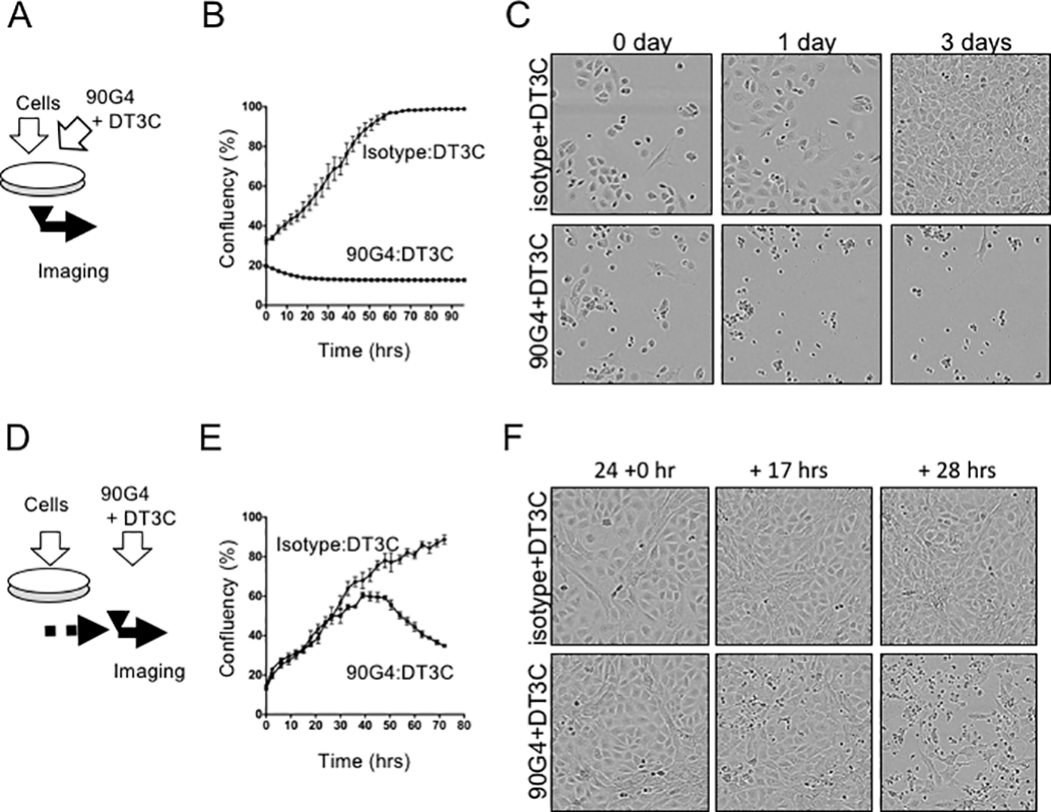
Targeted cytotoxicity of the 90G4 immunotoxin in migratory cells. Live imaging of 90G4 immunotoxin administrated either (A–C) concomitantly or (D–F) one day after SVEC4-10 cell seeding. For each condition, samples were seeded in triplicate. (B, E) Phase contrast images were taken every 3 h and analyzed to calculate confluency (%). Values and error bars correspond to mean and standard error, respectively. (C, F) Images were taken at the indicated time during live imaging.

CD321 is a junctional adhesion molecule that regulates proliferation, migration, and polarity in lymphocytes and endothelial cells [16]. To define the point of action of the 90G4 immunotoxin in endothelial cells, live imaging was employed. As shown in Fig 3A, SVEC4-10 cells and immunotoxin were seeded simultaneously. 90G4 immunotoxin but not isotype control caused immediate cell death after seeding, suggesting an important role for CD321 in endothelial cell proliferation (Fig 3B and 3C and S1 and S2 Videos). 90G4 antibody alone did not induce cytotoxicity. To investigate the effect on angiogenesis, we administered 90G4 immunotoxin one day after cell seeding (Fig 3D). Live imaging revealed an initial increase and then decrease in cell confluency (Fig 3E). Interestingly, the 90G4 immunotoxin initiated selective cytotoxicity against migratory endothelial cells rather than those forming sheets (Fig 3F and S3 and S4 Videos). Given that DT3C needs to be internalized to cause cytotoxicity, these results imply that migratory endothelial cells can actively internalize CD321 besides using it for tight junction formation. Accordingly, the 90G4 immunotoxin has potential antitumor activity by inhibiting de novo tumor angiogenesis.

### Redistribution of CD321

**Fig 4:**
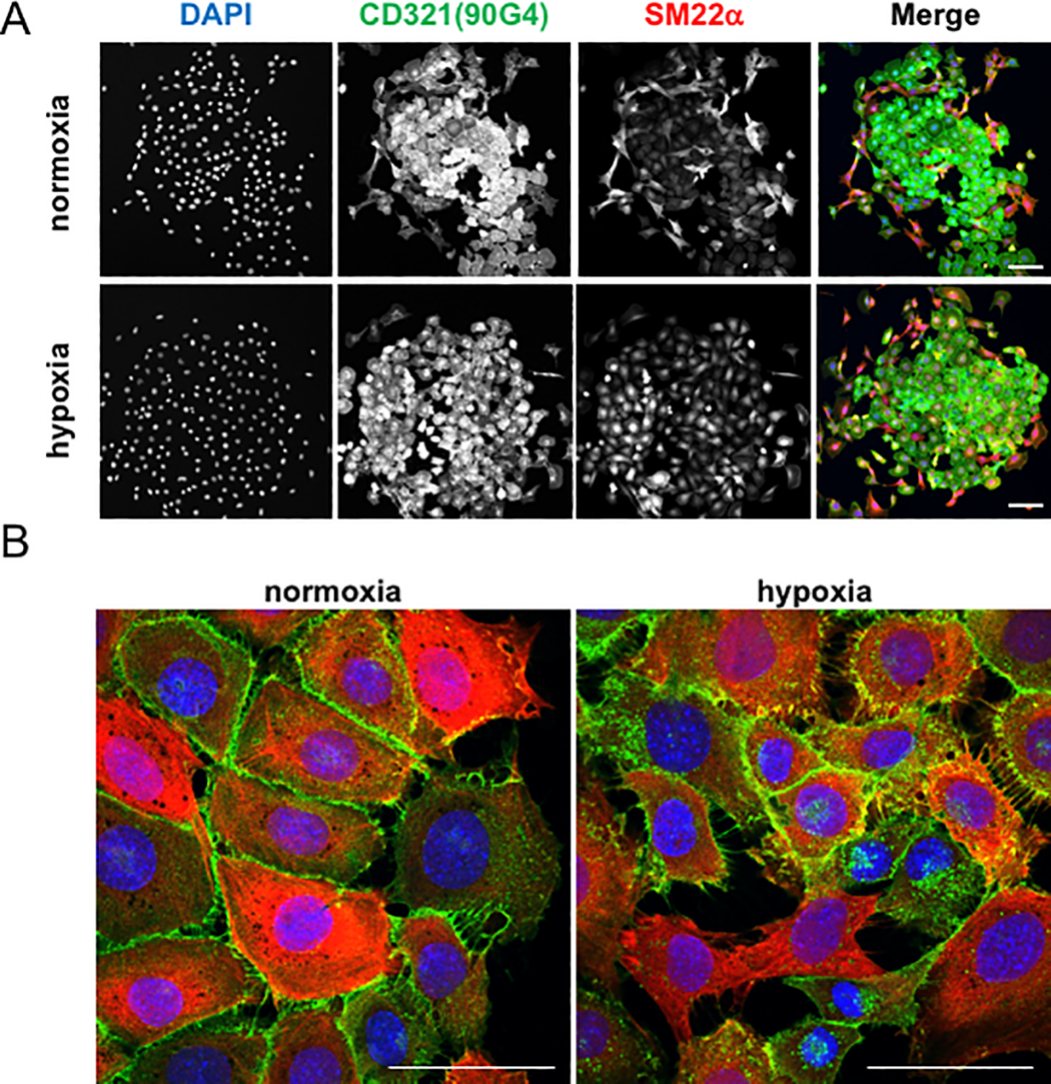
Redistribution of CD321 during hypoxia. Immunocytochemical analysis of CD321 localization. SVEC4-10 cells were incubated under normoxic (pO_2_ = 20%) or hypoxic (pO_2_ = 1%) conditions for three days. Cells were fixed and stained with DAPI (blue), 90G4 antibody (green), and anti-SM22α antibody (red). Images were taken at (A) low or (B) high magnification. Scale bar; 50 μm.

Increasing evidence supports the existence of triggering factors of metastasis, such as hypoxia, low pH, and nutrient starvation in the tumor microenvironment. We investigated whether CD321 responded to hypoxic stimuli by culturing SVEC4-10 cells under either normoxic or hypoxic conditions for three days. Under normoxia, the 90G4 antibody stained most of the edges of square-shaped cells (Fig 4A upper panel, center area), whereas the isotype control antibody did not give any signal (data not shown). As reported previously, 90G4 antibody staining localized at tight junctions. On the contrary, under hypoxic conditions, the 90G4 signal at tight junctions was less pronounced. Instead, a punctate signal was detected in the perinuclear region (Fig 4A lower panel). Redistribution appeared higher during hypoxia than normoxia and the redistributed CD321 signal was frequently observed in cells located at the periphery of a colony. This result may suggest hypoxia-induced redistribution. Although it cannot be explained at present, there seems to be a negative correlation between 90G4 antibody signal intensity at the cell surface and morphological changes from a rectangular to a rhombic cell shape.

We and other groups have reported previously that endothelial cells can differentiate into mesenchymal cells upon exposure to transforming growth factor-β (TGF-β) [10,17]. It is possible that in the present study a small population of endothelial cells was stimulated by TGF-β in the culture medium. A characteristic phenotype of mesenchymal differentiation is a change in cell shape and the expression of protein markers such as the transgelin SM22α. To this effect, multi-color staining with an anti-SM22α antibody revealed that high expression of SM22α often correlated with redistribution or loss of signal by the 90G4 antibody (Fig 4B). However, SM22α expression was not necessarily essential for redistribution of 90G4. In the present study, we could not address the detailed processes of redistribution due to technical limitations. We hypothesize that hypoxia partially enhanced redistribution of CD321.

To describe the precise localization of CD321 in the perinuclear regions, Z-stack images were taken by confocal laser microscopy at high magnification. Orthogonal projections revealed that during hypoxia, the 90G4 antibody localized either to the perinuclear region (excluding the nucleus) or the basal side of the cellular membrane (S2 Fig).

## Conclusion

In this study, we aimed to establish antibodies suitable for drug delivery to endothelial cells. Using DT3C-based hybridoma screening, we successfully generated the 90G4 antibody, which caused cytotoxicity in endothelial cells in the form of a DT3C immunotoxin (Fig 1). 90G4 antigen was widely expressed in several endothelial cell types (Fig 2A). Molecular analysis revealed that 90G4 recognized CD321, a junctional adhesion protein (Fig 2C and 2D). In comparison to control samples, live imaging demonstrated that the CD321 immunotoxin was actively internalized during cell proliferation and migration (Fig 3). Our findings describe the distinct roles played by CD321 in angiogenesis and tight junction formation.

We found that hypoxia led to the redistribution of CD321 to the basal side of endothelial cells, suggesting the initiation of functional modulation in these cells. It has been reported that in human endothelial cells, pro-inflammatory cytokines stimulate the insertion of CD321 in the luminal side, leading to the initial step of platelet deposition during atherogenesis [18]. Previous reports illustrated the redistribution of CD321 following basic fibroblast growth factor (bFGF) stimulation in starved HUVEC cells [19] and monocyte chemoattractant protein-1 (CCL2)/lipopolysaccharide (LPS) stimulation in mouse brain endothelial cells [20]. Stamatovic et al. (2012) demonstrated that micropinocytosis regulated the temporal redistribution of surface CD321 to the apical membrane within 10 to 20 min of LPS stimulation, suggesting that the inflammatory response in endothelial cells.

Our data suggest that CD321 internalization accelerated during hypoxia, but we could not provide quantitative results due to technical limitations. Although it is unclear whether redistribution of CD321 was primarily induced by hypoxia, it is worth noting that the large number of cells displaying relocated or diminished CD321 signal were positive for SM22α, a marker of mesenchymal differentiation. Endothelial-to-mesenchymal transition is known as the key event leading to functional impairment of endothelial cells in the tumor microenvironment, so the regulation of CD321 localization may be important.

CD321 is a potential target for diagnosis and therapy. It has been reported that soluble CD321 in serum or plasma is considered a biomarker of several diseases [21–24]. Moreover, several reports showed that anti-CD321 (clone BV11) antibody inhibited exaggerated skin inflammation [25] or experimental meningitis [26,27] via infiltration of inflammatory cells into the tissue. Since the 90G4 antibody possesses internalization activity that is suitable and functional for drug delivery, biodistribution of 90G4 antibody in vivo need to be validated in the future study. Although the selection of functional antibodies is still challenging, employing superior screening systems, such as the one we utilized in this study, provides promising candidate antibodies for antibody therapeutics.

## Supporting information

S1 Video 90G4 immunotoxin leads to cytotoxicity in proliferating cells. Live imaging was performed with immunotoxin (100 ng per well) of isotype control antibody.
(MP4)

S2 Video 90G4 immunotoxin leads to cytotoxicity in proliferating cells. Live imaging was performed with immunotoxin (100 ng per well) of 90G4 antibody.
(MP4)

S3 Video 90G4 immunotoxin leads to cytotoxicity in migratory cells. Live imaging was performed with immunotoxin (100 ng per well) of isotype control antibody.
(MP4)

S4 Video 90G4 immunotoxin leads to cytotoxicity in migratory cells. Live imaging was performed with immunotoxin (100 ng per well) of 90G4 antibody.
(MP4)

S1 Fig Immunocytochemical analysis of immunoreactivity of 90G4 antibody against other JAMs. Mouse CD322 (A) or CD323 (B) expression vector was transfected into CHO cells for 3 days, followed by fixation and permeabilization with 4%PFA and 0.05% Tween 20. Dual staining using 90G4 antibody (green) and anti-myc antibody (red) as primary antibodies, followed by secondary antibody staining with Alexa488-conjugated Donkey anti-Rat IgG (H+L) and Alexa555-conjugated Donkey anti-Mouse IgG(H+L) antibody, respectively. Nucleus was stained with DAPI. Scale Bar; 50 μm.
(TIF)

S2 Fig Orthogonal projection of CD321 localization. Orthogonal projections were generated from Z-stack images following 90G4 antibody staining. SVEC4-10 cells subjected to (A) normoxia or (B) hypoxia for three days. Scale bar; 50 μm.
(TIF)
