# Supplementary figures and images for: A novel immunotoxin reveals a new role for CD321 in endothelial cells
Source: PLoS One. 2017 Oct 13;12(10):e0181502. doi: 10.1371/journal.pone.0181502 (PMC5640210; doi:10.1371/journal.pone.0181502)

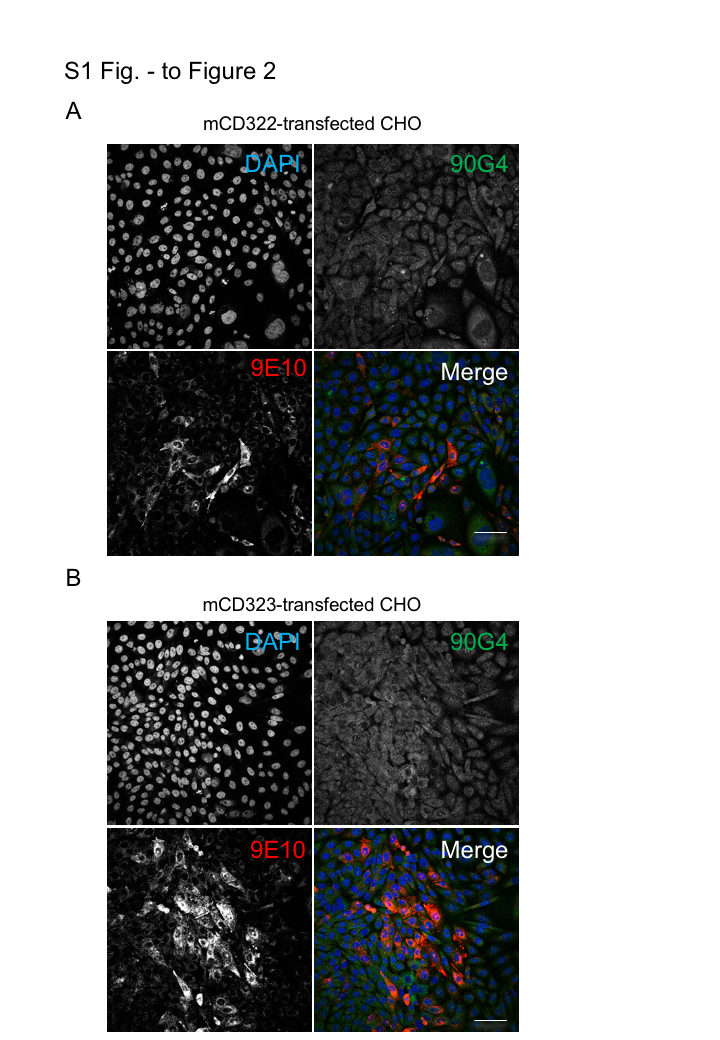

Supplement: S1 Fig — Mouse CD322 (A) or CD323 (B) expression vector was transfected into CHO cells for 3 days, followed by fixation and permeabilization with 4%PFA and 0.05% Tween 20. Dual staining using 90G4 antibody (green) and anti-myc antibody (red) as primary antibodies, followed by secondary antibody staining with Alexa488-conjugated Donkey anti-Rat IgG (H+L) and Alexa555-conjugated Donkey anti-Mouse IgG(H+L) antibody, respectively. Nucleus was stained with DAPI. Scale Bar; 50 μm. (TIF) [file pone.0181502.s005.tif]

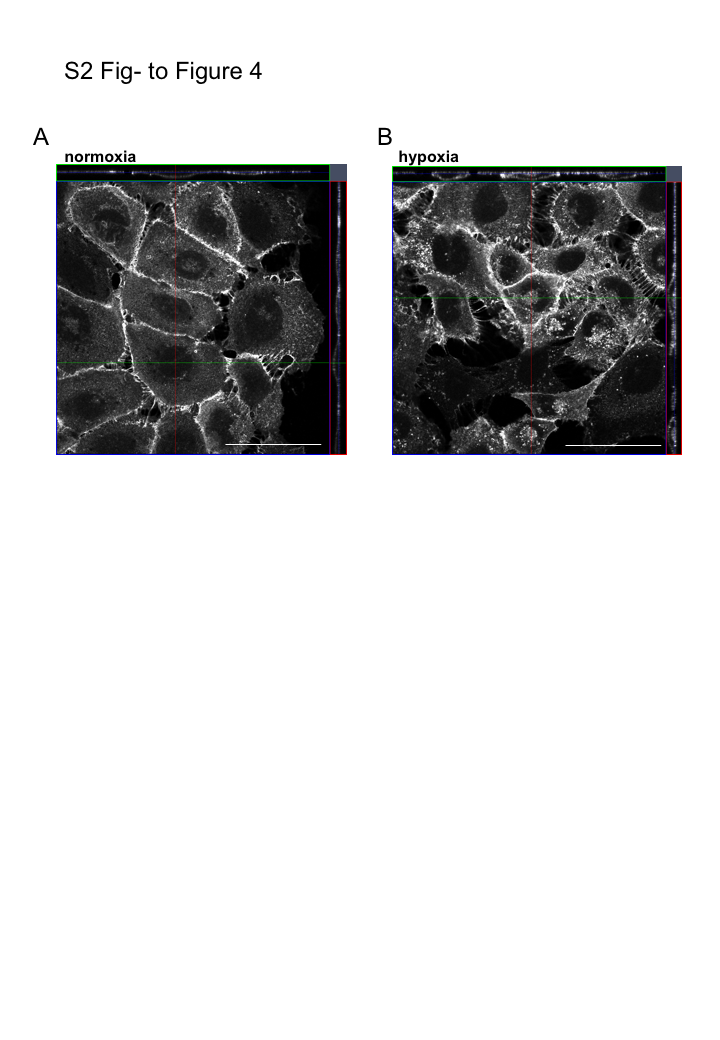

Supplement: S2 Fig — Orthogonal projections were generated from Z-stack images following 90G4 antibody staining. SVEC4-10 cells subjected to (A) normoxia or (B) hypoxia for three days. Scale bar; 50 μm. (TIF) [file pone.0181502.s006.tif]
